# Multifactorial Remodeling of the Cancer Immunopeptidome by IFNγ

**DOI:** 10.1158/2767-9764.CRC-23-0121

**Published:** 2023-11-17

**Authors:** Alice Newey, Lu Yu, Louise J. Barber, Jyoti S. Choudhary, Michal Bassani-Sternberg, Marco Gerlinger

**Affiliations:** 1 Barts Cancer Institute, Queen Mary University of London, London, United Kingdom.; 2 The Institute of Cancer Research, London, United Kingdom.; 3 The Proteomics Core Facility, The Institute of Cancer Research, London, United Kingdom.; 4 Department of Oncology UNIL CHUV, Ludwig Institute for Cancer Research, University of Lausanne, Lausanne, Switzerland.; 5 St Bartholomew's Hospital Cancer Centre, London, United Kingdom.

## Abstract

IFNγ alters the immunopeptidome presented on HLA class I (HLA-I), and its activity on cancer cells is known to be important for effective immunotherapy responses. We performed proteomic analyses of untreated and IFNγ-treated colorectal cancer patient-derived organoids and combined this with transcriptomic and HLA-I immunopeptidomics data to dissect mechanisms that lead to remodeling of the immunopeptidome through IFNγ. IFNγ-induced changes in the abundance of source proteins, switching from the constitutive to the immunoproteasome, and differential upregulation of different HLA alleles explained some, but not all, observed peptide abundance changes. By selecting for peptides which increased or decreased the most in abundance, but originated from proteins with limited abundance changes, we discovered that the amino acid composition of presented peptides also influences whether a peptide is upregulated or downregulated on HLA-I through IFNγ. The presence of proline within the peptide core was most strongly associated with peptide downregulation. This was validated in an independent dataset. Proline substitution in relevant core positions did not influence the predicted HLA-I binding affinity or stability, indicating that proline effects on peptide processing may be most relevant. Understanding the multiple factors that influence the abundance of peptides presented on HLA-I in the absence or presence of IFNγ is important to identify the best targets for antigen-specific cancer immunotherapies such as vaccines or T-cell receptor engineered therapeutics.

**Significance:**

IFNγ remodels the HLA-I–presented immunopeptidome. We showed that peptide-specific factors influence whether a peptide is upregulated or downregulated and identified a preferential loss or downregulation of those with proline near the peptide center. This will help selecting immunotherapy target antigens which are consistently presented by cancer cells.

## Introduction

The presentation of peptides on HLA class I (HLA-I) is central for the adaptive immune system to detect malignant cells. Presentation of immunogenic peptides such as nonmutated cancer-associated antigens or neoantigens on malignant cells facilitates their recognition and destruction by cytotoxic CD8 T cells. IFNγ is a cytokine that is released from activated CD8 T cells and other immune cell types. IFNγ binds to IFNγ receptors, which activate the JAK/STAT pathway, leading to expression of interferon response factor (IRF) transcription factors. IRFs stimulate the expression of a plethora of IFNγ-regulated genes leading to major changes in the cellular transcriptome and proteome (1). Proteins involved in the processing and subsequent presentation of peptide antigens on HLA-I molecules show particularly strong upregulation (2, 3), including the immunoproteasome catalytic components PSMB8, PSMB9, and PSMB10 which facilitate an increase in overall proteasomal activity, and also a specific increase of its chymotryptic activity (4–7). Peptidases which can trim, but also destroy, peptides before loading onto HLAs, such as LAP3 (8), THOP1 (9), ERAP1 (10), and ERAP2 (11), and the peptide transporters TAP1 and TAP2, which shuttle peptides into the endoplasmic reticulum where HLA loading occurs, are also upregulated. Furthermore, IFNγ increases HLA expression (6, 12). The combined result of increased proteasomal peptide generation, peptide processing and transport, and HLA upregulation, is a strong increase of peptide presentation by HLA-I on the cell surface. Further to this, IFNγ exposure inhibits the cell cycle and triggers apoptosis (13).

In contrast to these antitumor effects, IFNγ also promotes the expression of immunosuppressive molecules. These include PD-L1, the ligand of the PD1 immune checkpoint, and IDO1, whose expression in cancer cells and other cells in the tumor microenvironment suppresses T-cell activity (14–16). Immunotherapy with PD1/PD-L1 inhibitors has been highly successful in several cancer types (17–19). This supports a dominant role of the PD1/PD-L1 immune checkpoint in restraining tumor-reactive T cells. Consistent with a central role of IFNγ for PD-L1 expression, tumors that respond to PD1/PD-L1 inhibitors often show high IFNγ activity (20). Moreover, several recent studies have shown that defective IFNγ signaling in cancer cells leads to resistance to immunotherapy with checkpoint inhibitors (21–23).

Although intact IFNγ signaling in cancer cells is critical for checkpoint inhibitor efficacy, it is still unclear which specific IFNγ-induced molecular changes are responsible for this dependency. Understanding how the immunopeptidome is remodeled by IFNγ in greater detail may provide insights into this. Furthermore, novel immunotherapies such as cancer vaccines (24, 25) and engineered T-cell receptor (TCR)-based therapies such as Tebentafusp (26), target T cells toward specific peptide antigens presented on HLA of cancer cells. Understanding the characteristics of antigens that are consistently presented in the presence or absence of IFNγ, and which ones are lost or sparsely presented in one of these conditions, hence appears highly relevant for the selection of optimal target antigens.

Previously, we studied the immunopeptidome of five colorectal cancer patient-derived organoids (PDO) by mass spectrometry (MS). PDO cells were grown to large numbers followed by immunoaffinity capture of HLA-I–peptide complexes, analysis by high performance liquid chromatography and tandem MS (LC/MS-MS). This detected between 2,124 and 16,030 HLA-I peptides per PDO (27). Treatment of PDOs with IFNγ strongly increased HLA-I expression (mean 219.5% increase) but only had a modest effect on the number of unique peptides presented (mean 7.1% increase). However, a much larger number of peptides changed in abundance, and between 1,439 and 3,942 peptides were gained, and 561 to 2,446 peptides were lost on individual PDOs through IFNγ. Furthermore, we found that peptides generated by chymotryptic-like cleavage activity were more likely to increase in abundance, which we attributed to the switch of the proteasome to immunoproteasome triggered by IFNγ signaling, yet this effect was small.

Other MS immunopeptidome analysis of breast (28), lung (29), ovarian cancer (30), and melanoma cell lines (31) that were treated with IFNγ showed similar remodeling with a large proportion of peptides presented in only untreated or IFNγ-treated cells. This could be explained in part by effects of IFNγ on gene or protein expression, differences in HLA allotype upregulation, and the switch to the immunoproteasome. Yet, a large unexplained variance remained, highlighting a limited understanding of the molecular mechanisms and peptide features that regulate peptide abundance on HLA-I in IFNγ conditions. The aim of this work was to dissect the mechanisms that lead to upregulation/downregulation or appearance/loss of specific peptides under IFNγ exposure. We combined global cellular proteomic analysis with our published transcriptomics and immunopeptidomics datasets (27) to first investigate the impact of transcript and protein abundance on immunopeptidome remodeling, and to subsequently analyze peptide regulatory mechanisms that are independent of source protein abundance. The insights from this study should ultimately lead to more accurate predictions of the immunopeptidome in cells exposed to IFNγ, information which could be valuable for cancer vaccine or TCR therapy designs.

## Materials and Methods

### Ethics

Human samples were obtained from clinical trial protocols which have been approved by the UK National Ethics Committee (Prospect C trial approval number: 12/LO/0914, Prospect R trial approval number: 14/LO/1812, FOrMAT trial approval number 13/LO/1274). All individuals provided written informed consent for sample donation and use for research.

### PDO Culture and Treatment

Established PDOs were expanded to large numbers (3.85 × 10^7^–1 × 10^8^ cells/pellet) in DMEM/F12 media with 20% FBS, 1X Glutamax, 100 units/mL penicillin/streptomycin, and 2% matrigel (Corning, catalog no. 356231). For treatment, cells were changed into fresh media supplemented with DMSO or 600 ng/mL IFNγ (R&D Systems, catalog no. 285-IF/CF) and incubated for 48 hours. Cells were harvested with TrypLE express (Thermo Fisher Scientific, catalog no. 12605010). PDOs were cultured identically for transcriptomic, proteomic, and flow cytometric analysis.

### RNA Sequencing

We reanalyzed our previously described RNA sequencing data (27, 32)**.**

### Tandem-mass-tag Proteomics

PDOs were cultured as described, washed twice with ice-cold PBS and snap-frozen before further processing. Cell pellets were lysed with SDC lysis buffer [1% sodium deoxycholate, 100 mmol/L triethylammonium bicarbonate (TEAB), 10% glycerol, 50 mmol/L NaCl] with Halt protease and phosphatase inhibitor cocktail (Thermo Fisher Scientific). Cell pellet samples were completely homogenized with probe sonication (EpiShear) for 15 seconds at 40% power with 1 second on and 1 second off, heated at 90°C for 5 minutes and then repeated the probe sonication. Proteins were quantified using Quick Start Bradford Protein Assay (Bio-Rad).

A total of 100 µg protein was taken from each sample and lysis buffer was added so each sample was at the same volume. Proteins were reduced with 10 mmol/L tris(2-carboxyethyl)phosphine hydrochloride solution (Sigma) at room temperature for 10 minutes and then alkylated with 5 mmol/L iodoacetamide (Sigma) for 30 minutes at room temperature. Protein was then purified by 20% trichloroacetic acid precipitation. The pellet was resuspended in 100 mmol/L TEAB buffer, and digested by 3.3 µg trypsin (Pierce, MS Grade) at a ratio of 1:30 (trypsin:protein by weight) at 37°C for 18 hours.

A total of 40 µg of protein digest were labeled with 0.5 mg TMTpro 16plex reagents (Thermo Fisher Scientific) according to the manufacturer's instruction. After 1 hour incubation at room temperature and 15 minutes quenching by 4 µL of 5% hydroxylamine (Thermo Fisher Scientific), the labeled samples were combined. Sodium deoxycholate was precipitated by adding formic acid (FA; Honeywell Fluka). After centrifugation, the supernatant was collected and dried in Speedvac.

The sample were resuspended in 0.1% NH_4_OH/100% H_2_O, and fractionated on an XBridge BEH C18 column (2.1 mm i.d. × 150 mm, Waters) with an initial 5 minutes loading then linear gradient from 5% ACN/0.1% NH_4_OH (pH 10) to 35% CH_3_CN /0.1% NH_4_OH in 30 minutes, then to 80% CH_3_CN /0.1% NH_4_OH in 5 minutes and stayed for another 5 minutes. The flow rate was at 200 µL/minute. Fractions were collected at every 42 seconds from retention time at 7.8 to 50 minutes and then concatenated to 28 fractions and dried in SpeedVac. Samples were then resuspended in 0.5% FA for LC/MS-MS analysis. The protein abundance values in each sample were normalized by the loading input of each sample.

### LC/MS-MS Analysis

The LC/MS-MS analysis was on the Orbitrap Fusion Lumos mass spectrometer coupled with U3000 RSLCnano UHPLC system. All instrument and columns used below were from Thermo Fisher Scientific.

Fifty percent of peptides were injected. The peptides were first loaded to a PepMap C18 nanotrap (100 µm i.d. x 20 mm, 100 Å, 5 µm) at 10 µL/minute with 0.1% FA/H_2_O, and then separated on a PepMap C18 column (75 µm i.d. x 500 mm, 100 Å, 2 µm) at 300 nL/minute with a linear gradient of 8%–32% ACN/0.1% FA in 90 minutes/total cycle time at 120 minutes for each fraction. The data acquisition used standard data-dependant acquisition mode with a cycle time at 3 seconds. The full MS scans (m/z 375–1,500) were acquired in Orbitrap with a resolution at 120,000 at m/z 200, and the automatic gain control (AGC) was set at 400,000 with maximum injection time at 50 ms. The most abundant multiply charged ions (2+ to 5+) with intensity threshold at 5,000 were isolated by quadrupole at the isolation window at 0.7 Da and then subjected to MS-MS fragmentation by collision-induced dissociation in ion trap at 35% normalized collision energy (NCE). The AGC was set at 10,000 and maximum injection time at 35 ms. The tandem-mass-tag (TMT) report ions were detected by further fragmentation of the five most abundant fragment ions produced in MS2: they were isolated by synchronous precursor selection (SPS) method with the isolation width at 0.7 Da, and fragmented by higher energy collisionally activated dissociation at 55% NCE, and detected in the Orbitrap in a scan range 100–500 m/z. The resolution was set at 50,000 at m/z 200, the AGC at 50,000 with maximum injection time at 86 ms. The dynamic exclusion was set 40 seconds with ± 10 ppm exclusion window.

### Mass Spectral Data Processing

All raw files were processed in Proteome Discoverer 2.4 (Thermo Fisher Scientific) using the Sequest HT search engine to searched against reviewed Uniprot database of Homo Sapiens (Version February 2020) and contaminate database (from Thermo Fisher Scientific). Search parameters were: trypsin with two maximum missed cleavage sites, mass tolerances at 10 ppm for the precursor, and 0.5 Da for the fragment ions; dynamic modifications of Carbamidomethyl (C), Deamidated (N, Q), TMTpro (K, peptide N-terminus) and Oxidation (M), and Acetyl (protein N-terminus). Search result was validated by Percolator with *q*-value set at 0.01 for the decoy database search, and only high confident PSMs (Peptide Spectrum Matches) were considered. Protein FDR was set at 0.01. Only master proteins were reported. For reporter ion intensity detection, the reporter ion quantifier node parameters were integration window tolerance 20 ppm, integration most confident centroid for peak detection. Only unique peptides were considered for quantification. TMTpro Quan value correction factor, provided by the manufacturer's certificate of analysis, was applied. Coisolation threshold was set at 100, reporter ions average S/N threshold at 3 and SPS mass matches threshold 55%. Report ions intensities were normalized by total peptide amount to correct the variation by for different protein loading in each channel, and then scaled on all average.

### MS Immunopeptidomics

MS immunopeptidomics data had been acquired as described previously (27, 30). The individual peptide intensity values in the IFNγ samples were divided by the fold change (FC) in total peptide intensity between untreated and IFNγ-treated conditions for each PDO. This normalized for the change in HLA-I expression in the IFNγ-treated condition, which led to broadly increased peptide intensities. This enabled comparison of relative peptide intensities between untreated and IFNγ-treated conditions. For the analysis of the amino acid composition of peptides that are upregulated or downregulated we further normalized peptide intensities within each HLA allotype; intensity of each of the IFNγ peptides was divided by the FC in total peptide intensity for that allotype between untreated and IFNγ-treated conditions. The validation dataset was obtained from (28) and was normalized in the same way.

### Prediction of MS-detected Peptide-HLA-I Affinity with NetMHCpan

All MS-detected HLA-I peptides were entered into NetMHCpan4.1b, with standard settings [strong binders defined as binding affinity (BA) rank ≤0.5% and weak binders as 0.5%–2% BA rank] (33). Each peptide was then assigned to the HLA allotype with the lowest rank, and this was used to subset peptides in to HLA-I–allocated groups.

### Prediction of MS-detected Peptide–HLA-I Complex Stability with NetMHCstabpan

All MS-detected HLA-I peptides were entered into NetMHCstabpan1.0 with standard settings (strong binders defined as rank ≤0.5% and weak binders as 0.5%–2% rank) to obtain the predicted peptide-HLA half-life.

### Relative Peptide Start Position

Relative peptide start position within protein was calculated for each peptide by dividing the peptide start position by the full protein length. The longest protein length for each protein was selected from the MS database search Fasta file to ensure every peptide was encompassed. Relative start position was assigned from zero indicating the first translated amino acid.

### Absolute Peptide Start Position

The peptide absolute start position in the protein was derived from the MaxQuant output.

### HLA Typing

HLA typing results from the previous publication were used (27).

### Surface HLA Quantification by Flow Cytometry

HLA surface expression was assessed using pan-HLA-A/B/C antibody [Clone: W6/32, RRID: AB_314871 (BioLegend, catalog no. 311402)], anti-HLA-A03 [Clone: GAP.A3, RRID: AB_2572503 (Thermo Fisher Scientific, catalog no 11-5754-42)], and anti-HLA-B27 [Clone: HLA-ABC-m, RRID: AB_322098 (Bio-Rad, catalog no. MCA116F)]. Samples were run on a Sony SH800 cell sorter.

### Statistical Analysis

Statistical calculations and plots were performed in R (www.r-project.org) and on GraphPad Prism v9 (GraphPad Prism, RRID:SCR_002798). Z-scores for the amino acid enrichment analysis were calculated by subtracting each value by the mean of all the difference values, then dividing by the SD of all the difference values.

### Data Availability

RNA sequencing data were published in the Supplementary Materials and Methods to ref. 32. Processed immunopeptide, RNA and proteome data for each of the three PDOs are provided as Supplementary Data.

The MS immunopeptidomics data were deposited to the ProteomeXchange Consortium via the PRIDE (34) partner repository with the dataset identifier PXD014017, and the global proteomics dataset with the dataset identifier PXD031634.

### Ethics

Human samples were obtained from clinical trial protocols which have been approved by the UK National Ethics Committee (Prospect C trial approval number: 12/LO/0914, Prospect R trial approval number: 14/LO/1812, FOrMAT trial approval number 13/LO/1274). All individuals provided written informed consent for sample donation and use for research.

## Results

**FIGURE 1:**
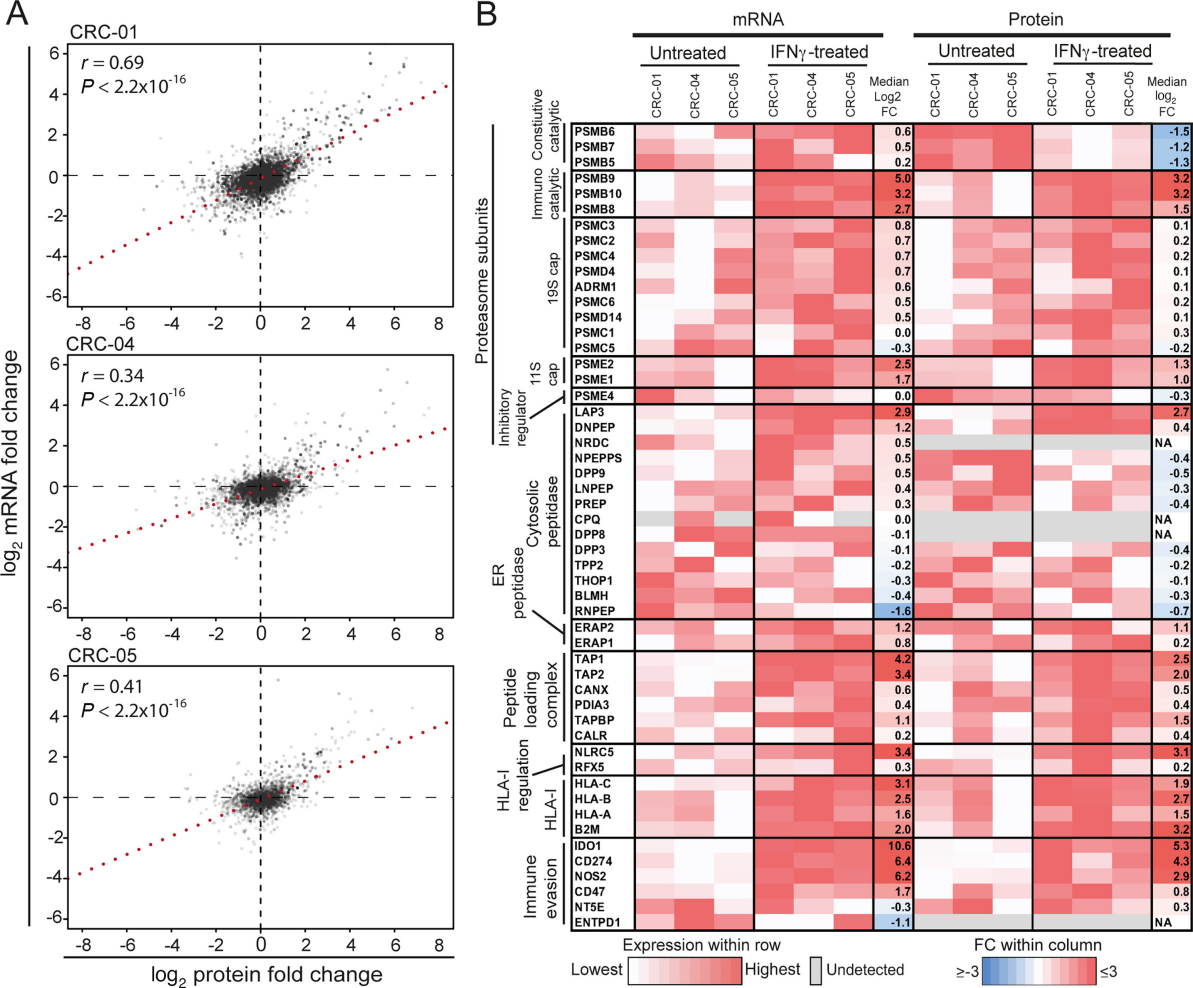
Transcriptomic and proteomic changes with IFNγ treatment*.***A,** Correlation of the FC in normalized mRNA read numbers against the FC in normalized protein intensity. The Spearman rank test was used for statistical analysis. **B,** mRNA expression and protein intensity of selected genes in untreated and IFNγ conditions.

The aim of this study was to elucidate the molecular mechanisms through which IFNγ alters HLA-I peptide presentation by comparing the immunopeptidome of untreated and 48-hour IFNγ-treated colorectal cancer PDOs (CRC-01, CRC-04, CRC-05). This was achieved by combining previously generated transcriptomics and immunopeptidomics data (27) with new global proteomics data, obtained by TMT-MS. A total of 7,408 proteins were detected by TMT-MS across the three PDOs. IFNγ-induced FC of transcript and protein abundance (Fig. 1A) showed a significant positive correlation (Spearman *r* = 0.34–0.69, *P* < 2.2 × 10^−16^ for all three PDOs). IFNγ increased the expression of a large number of transcripts/proteins whereas downregulation was only apparent in a smaller number of transcripts/proteins and was of limited magnitude.

We next assessed whether transcripts/proteins that were previously described as IFNγ-regulated, and have roles in antigen processing and presentation or immune evasion, undergo the expected changes. IFNγ treatment increased RNA expression of most proteasome components, including constitutive proteasome catalytic subunits (PSMB5–7), and immunoproteasome catalytic components (PSMB8–10; Fig. 1B). In contrast, proteome data showed a strong decrease of the constitutive catalytic subunits. This disparity between RNA and protein abundance can be explained by the fact that immunoproteasome assembly is four times faster than that of the constitutive proteasome (35), so the excess unbound constitutive catalytic subunits will be degraded (4). This switch from constitutive to immunoproteasome alters the cleavage specificity toward an increased chymotryptic activity, as we observed in our previous study (27). The regulatory caps of the proteasome also change with IFNγ treatment; in the absence of IFNγ, the 26S proteasome forms by addition of the 19S cap to each end of the 20S proteasome core. The 19S cap is responsible for binding polyubiquitinated proteins and actively transporting them in to the 20S proteasome core. IFNγ increases the expression of the 11S cap subunits which facilitate ubiquitin-independent proteasomal degradation of proteins and enhance proteasomal throughput (36, 37). PSME4 is another proteasome cap subunit, recently shown to impede the production of HLA-I compatible peptides (38). The decrease in PSME4 protein abundance through IFNγ may further increase peptide production.

Among cytosolic peptidases, LAP3 (with a cleavage specificity toward hydrophobic N-terminal amino acids, primarily leucine) increased in protein abundance (Fig. 1B), and most other cytosolic peptidases showed a small decrease. Both endoplasmic reticulum N-terminal aminopeptidases ERAP1 and ERAP2, which help to shape the immunopeptidome by final trimming on HLA-I (10, 11), increased. TAP transporters and peptide loading complex components increased in RNA expression and protein abundance. Further to this, NLCR5, the master transcription factor for HLA-I expression, and consequently HLA-A, -B, and -C increased strongly.

We also assessed whether genes and proteins that are known to inhibit the activity of immune cells were upregulated by IFNγ. Most immune evasion genes increased in expression, with IDO1 and CD274/PD-L1 showing the strongest increase at the protein level. Thus, IFNγ triggered expected changes in known IFNγ-regulated genes across all three organoid lines.

**FIGURE 2:**
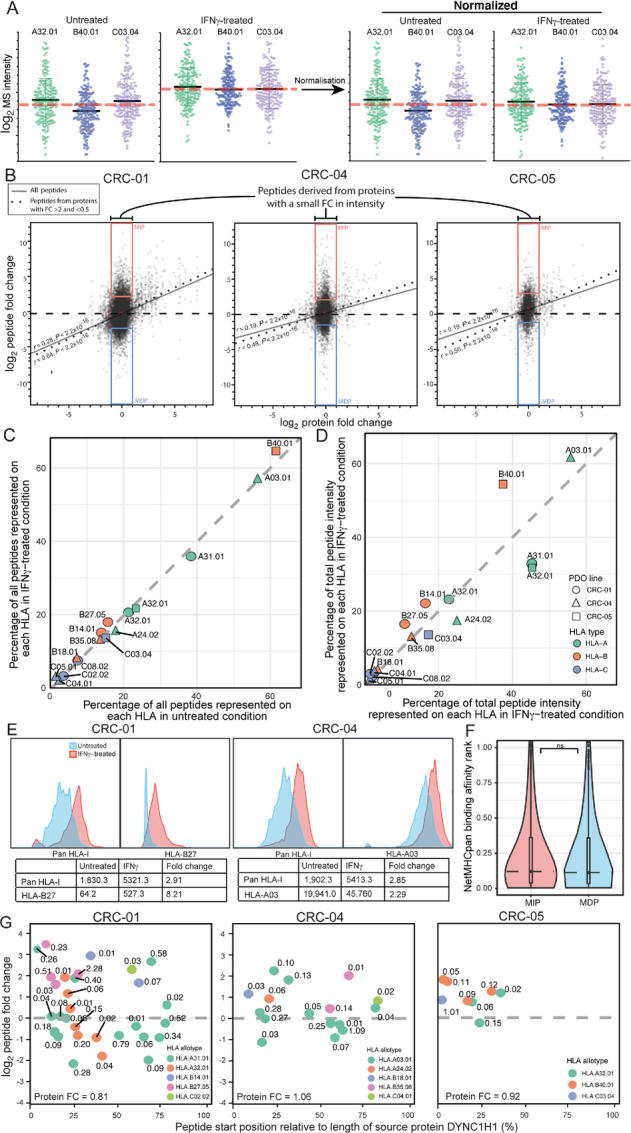
Influence of protein abundance and HLA expression changes on immunopeptidome remodeling. **A,** Schematic demonstrating the principle of the peptidomics normalization process with sampled data from CRC-05 which only has three HLA-I alleles: the total peptide intensities of untreated and IFNγ-treated sample pairs were normalized to be equal. This allowed comparison of how IFNγ treatment changes the abundance of individual peptides within the entire HLA-I–presented peptide population. Black line: mean peptide intensity for each HLA allotype, dotted red line: mean peptide intensity across all peptides per condition. **B,** Correlation of protein FC between untreated and IFNγ conditions, against normalized immunopeptidomics FC. Regression lines for all peptides are displayed as a solid black line, regression lines for peptides from proteins with FC <0.5 or >2 as a dotted line. The Spearman rank test was used for significance testing. MIPs (top 10th percentile peptide FC) and MDPs (bottom 10th percentile peptide FC) derived from low FC proteins (0.5–2× FC) are highlighted with red and blue boxes, respectively. **C,** Percentage of all peptides per PDO that were attributed to each HLA by NetMHCpan4.1 in untreated versus IFNγ-treated conditions. **D,** Percentage of the total peptide intensity per PDO represented on each HLA (attributed by NetMHCpan4.1) in untreated versus IFNγ-treated conditions. **E,** Expression of total surface HLA-I and single HLA-I allotypes in organoid lines CRC-01 (HLA-B27) and CRC-04 (HLA-A03), measured by flow cytometry. **F,** log_2_ NetMHCpan4.1-predicted BA ranks for MIPs versus MDPs. The median is marked with a dotted line. **G,** log_2_ change in peptide intensity between untreated and IFNγ-treated conditions for peptides derived from the protein DYNC1H1, plotted against the relative position of the peptide in the protein. Peptides are color coded by their NetMHCpan4.1-predicted source HLA, with the NetMHCpan4.1-predicted BA rank annotated above. The FC of the DYNC1H1 protein in each PDO is noted at the bottom.

We next assessed to what extent a change in abundance of source proteins influenced the presentation of their derived peptides on HLA-I. Normalization of the immunopeptidome data was performed similar to the normalization approach used for RNA and protein expression data: peptide intensity values were normalized so that the total intensity in untreated and IFNγ-treated conditions was identical within each PDO (schematic of normalization: Fig. 2A). This allowed us to investigate peptide abundance changes beyond those driven by the absolute increase in HLA-I expression with IFNγ. We subsequently plotted the FC in protein abundance between untreated and IFNγ-treated conditions against normalized FC of all HLA-I presented peptides (Fig. 2B). This analysis revealed two distinct components in each PDO: one group of upregulated or downregulated peptides which showed concordant changes in the source protein abundance through IFNγ, and a second group of upregulated or downregulated peptides derived from proteins with no or limited change in abundance (log_2_ −1 to 1 FC). This shows that an increase in source protein abundance, which increases availability for proteasomal breakdown, is one important driver of peptidome remodeling, but also that the surface presentation of an even larger number of peptides is controlled by additional mechanisms.

Our next aim was to understand how this second group of peptides is regulated in these PDOs. We first hypothesized that IFNγ upregulates different HLA alleles by different levels, and that this may affect the diversity or abundance of their corresponding peptide repertoires. Plotting the number of unique peptides predicted to bind each HLA allele by NetMHCpan4.1 (33), showed only a small change in the number of unique peptides presented on each HLA allele after IFNγ treatment (Fig. 2C). Next, we plotted the total peptide intensity per HLA allele as a surrogate measure of the total abundance of peptides presented on each HLA. When treated with IFNγ, the relative intensity of peptides presented on HLA-B increased, whereas those on three of five HLA-A decreased (Fig. 2D). Therefore, while absolute numbers of HLA-A, -B, and -C proteins and presented peptides increased based on the proteomics and non-normalized ligand data, our results showed that under IFNγ exposure the proportion of the entire peptidome expressed on HLA-A decreased, and that on HLA-B increased.

To validate these findings, we performed flow cytometry staining for total HLA-I and for two HLA allotypes (HLA-A03 and HLA-B27), for which specific mAbs were available. Pan-HLA-I antibody staining on CRC-01 showed a 2.91-fold upregulation of total HLA, whereas staining for HLA-B27 showed an 8.21-fold increase (Fig. 2E), 2.82-fold more than the total HLA. Further to this, CRC-04 showed a 2.85-fold upregulation of total HLA and 2.29-fold upregulation of HLA-A03, which is 0.8-fold that of the total HLA upregulation (Fig. 2E). This validated the results from the peptide analysis and is consistent with both historical transcriptional studies (39, 40), and more recent peptidomics studies (30, 41, 42), which showed a stronger upregulation of HLA-B compared with HLA-A molecules with IFNγ.

We next assessed whether binding affinities of peptides to their cognate HLA allotype may influence their upregulation or downregulation. We focused on 9-mer peptides that originated from source proteins with modest abundance changes (defined again as log_2_ −1 to 1 FC), yet changed strongly in intensity. We defined a strong change in intensity as the top and bottom 10th percentile of the peptidomics FC data [most increasing peptides (MIP) and most decreasing peptides (MDP)] in each of the three PDOs; MIPs highlighted with a red box, and MDPs with a blue box (Fig. 2B). Plotting the NetMHCpan4.1-predicted affinities of MIPs and MDPs for their HLA (Fig. 2F) revealed a similar data distribution between the two groups, and no significant difference between medians (*P* = 0.9708, Mann–Whitney test). Thus, peptide BA did not noticeably impact whether a peptide was upregulated or downregulated by IFNγ.

Our next hypothesis was that other peptide-specific factors determine upregulation or downregulation, independently of protein and HLA abundance changes. To assess this, we first focused on large proteins that each contributed multiple MS-detected peptides in individual PDOs. A single protein can undergo a specific change in abundance and turnover with IFNγ treatment and this should affect all peptides that originate from that protein similarly. We therefore reasoned that the detection of upregulation and downregulation of peptides from the same protein with IFNγ treatment would indicate that peptide-specific characteristics influence these abundance changes. One limitation of this approach is that it does not control for differences in protein isoforms, which may be relevant for some peptides. Analysis of DYNC1H1, the protein that contributed the largest number of peptides across each of our PDOs, (Fig. 2G), showed that some peptides originating from the same protein increased, whereas others decreased with IFNγ treatment. This appeared independent of the cognate HLA allotype, and is hence not the consequence of differential HLA-A and -B upregulation. Analysis of nine additional large proteins, showed similar results (Supplementary Fig. S1A–S1I). Thus, peptide-specific factors play a major role in determining whether a peptide is up or downregulated through IFNγ.

Some publications identified an overrepresentation of peptides derived from the N-terminus of a protein due to premature termination of translation or nonsense-mediated decay, but whether this effect increases or decreases with IFNγ treatment is unknown (43, 44). In the analysis of 10 long proteins, clustering of upregulated peptides close to the N-terminus was observed for DYNC1H1 in CRC-05, but no systematic increase or decrease in the abundance of peptides located closer to the N-terminus was apparent with other PDOs/proteins (Fig. 2G; Supplementary Fig. S1A–S1I).

To assess a much larger number of datapoints, we plotted the frequency of MIPs and MDPs against their absolute location in the source protein (Supplementary Fig. S1J). This showed only modest differences, suggesting that location within the protein had little effect on peptide production between untreated and IFNγ conditions, which agrees with another immunopeptidomics study (45).

**FIGURE 3:**
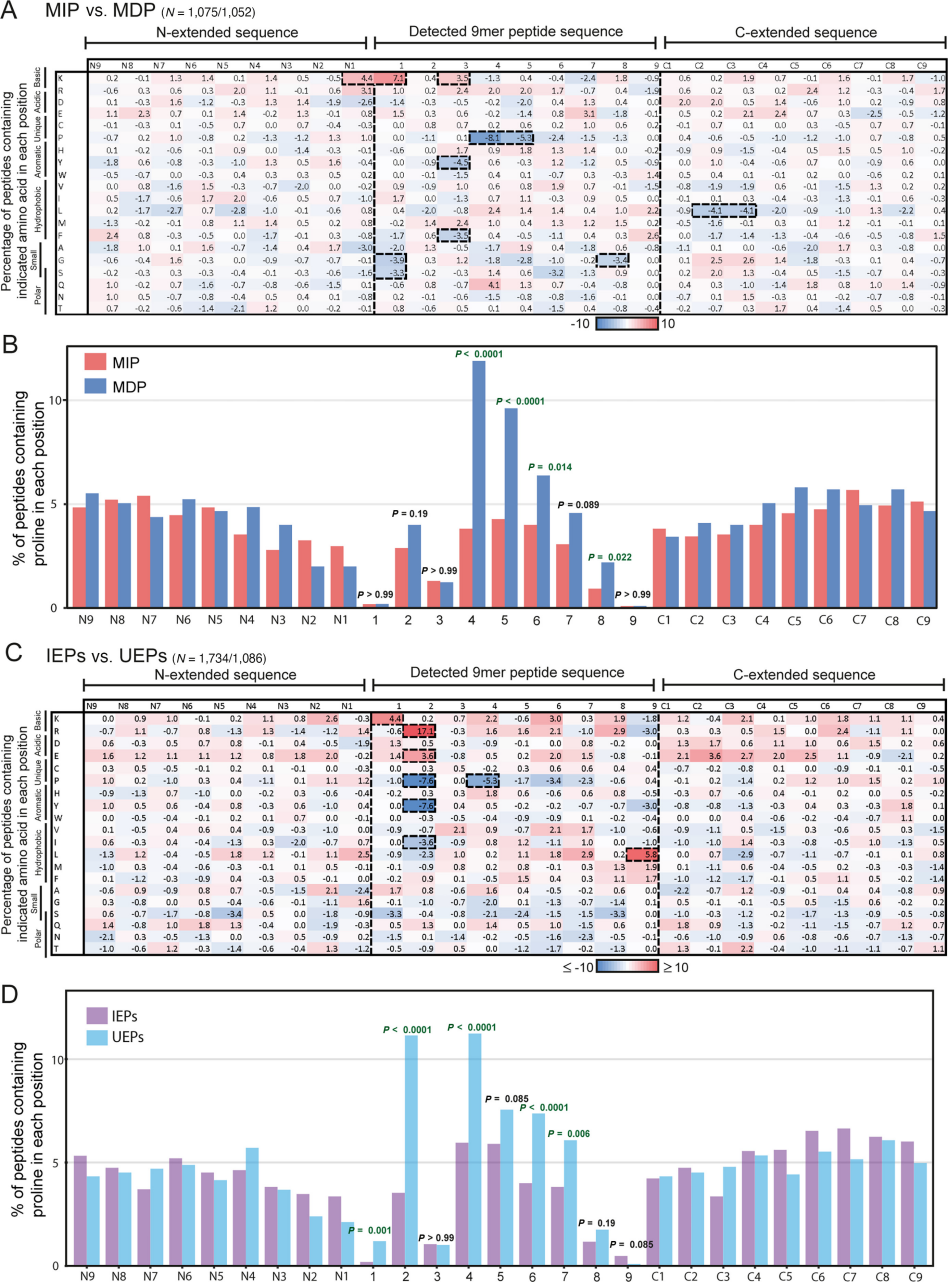
Amino acid composition of MIPs versus MDPs and UEPs versus IEPs. **A,** Heat map of the amino acid composition changes between 9-mer MIPs and MDPs, alongside N- and C extensions. Percentage of peptides with highlighted amino acid in each position were calculated for each group, then the percentage values for the MDPs (*N* = 1,052) were subtracted from the MIPs (*N* = 1,075). **B,** Percentage of peptides with proline in each position for MIPs (*N* = 1,075) and MDPs (*N* = 1,052). Fisher exact test was used for statistical analysis of the proline abundance values, with significant results are indicated in green**. C,** Heat map of the amino acid composition changes between (IEPs) and (UEPs). Percentage of peptides with each amino acid in highlighted position were calculated for each group, then the percentage values for the UEPs (*N* = 1,195) were subtracted from the IEPs (*N* = 1,909). **D,** Percentage of peptides with proline in each position in IEPs and UEPs. Z-score analysis was used for the heat maps, and changes with a Z-score ≤2.5 or >2.5 were highlighted with a thick dotted line. Fisher exact test was used for statistical analysis of the proline abundance values, with significant results are indicated in green.

We next investigated whether the amino acid composition of MIPs or MDPs influenced the IFNγ-induced changes in peptide abundance. To reveal peptide characteristics which are not simply a consequence of the differential upregulation of distinct HLA allotypes, peptides were first separated into those binding each specific HLA allotype based on NetMHCpan4.1 prediction. The peptide intensities for each allotype were then normalized so that the total intensity in untreated and IFNγ-treated conditions was identical for each allotype. Next, MIPs and MDPs (again defined as the top and bottom 10th percentile of the immunopeptidomics FC values) were selected individually for each HLA, and all upregulated peptides were then combined as the MIPs and all downregulated peptides as MDPs. Thus, the same number of upregulated and downregulated peptides were identified from each HLA. This approach of selecting within HLA allotypes can identify differences in the characteristics of MIP versus MDP peptides that are independent of the specific HLA and their peptide binding motifs. We then analyzed the frequency of all amino acids at each position of the 9-mers, and values for MDPs were subtracted from MIP values (Fig. 3A). The amino acid composition of the N-terminal and the C-terminal extensions adjacent to the presented peptide may also influence peptide processing (46), for example through the presence of specific cleavable amino acids or motifs for proteasome or peptidase processing. Therefore, we also assessed the 9aa N- and C-terminal extensions.

Most differences in amino acid abundance at each position of the 27aa sequence were small, but Z-score analysis identified 12 amino acids in specific positions where their difference in abundance was 2.5 times larger than the SD of all difference values (dotted outlines in Fig. 3A).

There was an overrepresentation of lysine, a basic amino acid, in position N1 of the N-terminal extension, with a Z-score of 3.4. Notable in this context was also the less marked overrepresentation of arginine, another basic amino acid, in position N1 (which fell below the Z-score cutoff, with a score of 2.4)—similar to findings which showed activity of immunoproteasomes is higher against basic residues in position N1 (7). Although the increase in basic residues in position N1 may suggest an increase in tryptic cleavage activity with IFNγ, we also identified an overrepresentation of lysine (Z-score of 5.6) in position 1 of MIPs. This cannot be explained by tryptic activity, as tryptic-like activity cleaves to the C-terminus of a basic residue, not the N-terminus. The small amino acids glycine and serine were underrepresented in position 1 of the MIPs (Z-scores of −3.0 and −2.6, respectively). This may be caused by the exchange of PSMB5, whose binding pocket has a preference for small amino acids, for PSMB8 in the immunoproteasome, which has a preference for chymotryptic-like substrates (5). However, some of these changes may also be due to activity of peptidases, as peptidases commonly cleave away additional amino acids from the N-terminus of a longer precursor peptide (46).

We also assessed whether the previously described increase in intensity of peptides with a chymotrypsin-like cleavage site at the C-terminus, which we attributed to the immunoproteasome switch (27), was apparent among MIPs. Three of the seven (42.3%) amino acids (A, F, I, L, M, V, or Y in position 9) which are preferentially cleaved by chymotrypsin increased in representation in MIPs, whereas only two out of 13 (15.4%) of the remaining amino acids at position 9, increased. The net increase of these seven amino acids after which chymotrypsin-like activity occurs was 1.8%. Peptides with trypsin-like cleavage sites (K or R in position 9) showed a net decrease of 2.8% which is consistent with a lower activity of the constitutive proteasome.

The only positions in the C-terminal extension highlighted by the Z-score analysis were positions C2 and C3, in which leucine was underrepresented (Z-score: −3.2 in both positions). The widely accepted view is that the C-terminus of the peptide is directly generated by the proteasome (30, 47), as no carboxypeptidases have been identified in antigen processing. Therefore, one possible explanation for our observations is that the leucines in C2 and C3 influence pepide generation by the immunoproteasome.

The amino acid with the largest difference within the 9-mer peptides was proline. This was underrepresented in positions 4 and 5 of MIPs with Z-scores of −6.3 and -4.2, respectively (Fig. 3A). Underrepresentation continued in the consecutive positions 6–8, but these did not cross the Z-score threshold (−1.9, −1.2, and −1.0, respectively). We next plotted the proline abundance in MIPs and MDPs for each position of the peptide and its N- and C-terminal extension and applied statistical testing. This showed significant differences for positions 4–6 and 8 (Fig. 3B). We furthermore analyzed all 10-mer MIP and MDP peptides identically to the 9-mers to ascertain whether these observations could be reproduced. This confirmed a similar underrepresentation in MIPs in positions 4–7, crossing the Z-score threshold in positions 4 and 5 (Supplementary Fig. S2A). To assess whether the depletion of proline in MIPs was detectable across PDOs and different HLA allotypes, we furthermore analyzed the proline abundance in each position of 9-mer peptides separately for each PDO and HLA allotype. This showed consistent proline underrepresentation in MIPs, most strongly in positions 4 and 5 (Supplementary Fig. S2B). Thus, peptides with proline in positions 4–5 were more likely to be downregulated through IFNγ treatment and this was neither a PDO, nor an HLA-specific effect. Prolines in position 6–8 appeared to have a similar, but less pronounced effect. The heat maps further demonstrated that as proline decreased in abundance, there was no corresponding increase in another amino acid, but small increases dispersed among several amino acids. This suggests that the decrease of proline is a specific effect of IFNγ.

We also detected other larger changes in Z-score within the peptide: −3.5, −2.7, and 2.8 for tyrosine, phenylalanine, and lysine in P3; and −2.7 for glycine in P8. However, these findings were not reproduced in the original positions, or original positions ±1 in the analysis of 10-mers (Supplementary Fig. S2A).

We next assessed whether peptides that were only detected in untreated PDOs (untreated-exclusive peptides—UEP) or only detected with IFNγ treatment (IFNγ-exclusive peptides—IEP), and derived from proteins with a low FC (log_2_ −1 to 1 FC), showed the same signal. Proline was again underrepresented at positions 4–7 in IEPs compared against the UEPs (Fig. 3C). The difference in proline abundance between UEPs and IEPs was significant in positions 4, 6, and 7 (Fig. 3D). Of note, we also observed changes in the peptide anchor positions 2 and 9 that had not been apparent in the MIP versus MDP analysis. This can be explained by the relative overrepresentation of peptides presented by HLA-B among IEPs and by HLA-A in UEPs, which is a consequence of the different levels of upregulation with IFNγ we described above. To control for this, we also separated the condition-exclusive peptide groups by their source HLA (Supplementary Fig. S2C). Because of the lower peptide numbers there is more variation in the data, but the IFNγ-exclusive peptides showed a decrease in proline abundance in position 4 across different HLA allotypes.

Taken together, our approach to scrutinize the changes in the peptidome showed that IFNγ treatment results in an increased production of peptides with lysine, and a decreased production of peptides with small amino acids, at the N-terminal position 1. Further to this, we found a decrease of peptides with proline in the peptide core, mainly positions 4 and 5, a pattern that was present across all three PDOs and different HLAs, strongly suggesting that this effect is conserved across biological models.

**FIGURE 4:**
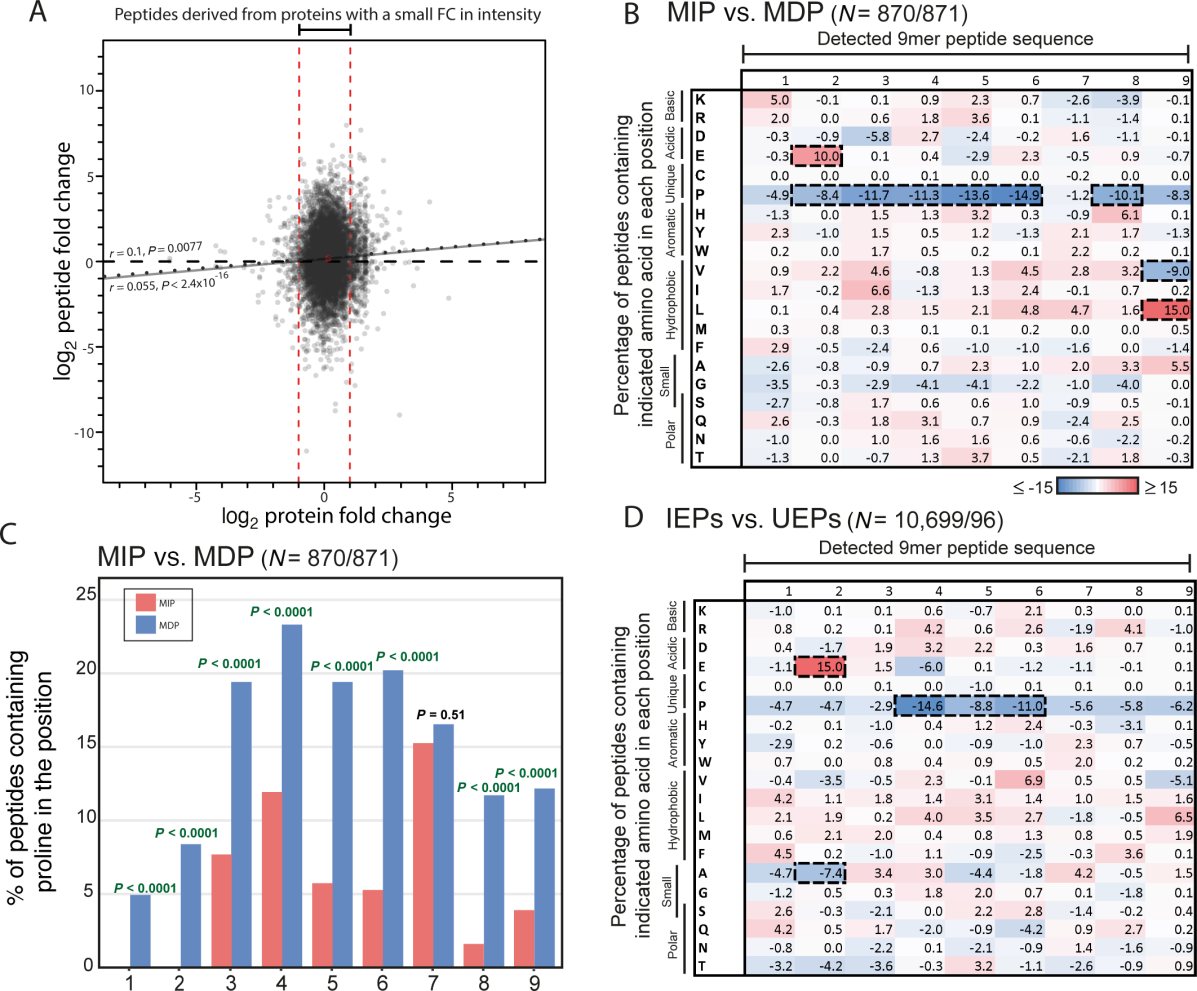
Validation of amino acid differences in the datasets from Goncalves and colleagues. **A,** Correlation of proteomics FC between untreated and IFNγ conditions, against normalized immunopeptidomics FC. Regression line for all peptides marked by a solid black line, regression line for peptides derived from proteins with FC > 2 and < 0.5 marked with a dotted line. Spearman rank analysis used to investigate correlation. Low FC proteins (0.5–2× FC) were marked out with red dotted lines. **B,** Heat map of the amino acid composition changes between 9-mer MIPs and MDPs, alongside N- and C-terminal extensions. Peptides were grouped by their NetMHCpan4.1-predicted HLA-I allotype, then from each group the top 10th percentile peptide FC (MIPs) and bottom 10th percentile peptide FC (MDPs) were selected. Detailed peptide numbers provided in Supplementary Table S1. Percentage of peptides with highlighted amino acid in each position was calculated for each group, then the percentage values for the MDPs were subtracted from the MIPs. **C,** A graph depicting the percentage of peptides with proline in each position for the MIPs and MDPs. **D,** Heat map of the 9-mer peptide amino acid constituent changes between (IEPs) and (UEPs). Peptides were selected from “low FC” proteins (0.5–2× FC). Percentage of peptides with highlighted amino acid in each position were calculated for each group, then the percentage values for the UEPs were subtracted from the IEPs. Detailed peptide numbers provided in Supplementary Table S2. Z-score analysis was used for the heat maps, and changes with a Z-score ≤2.5 or >2.5 were highlighted with a thick dotted line. Fisher exact test was used for statistical analysis of the proline abundance values, with significant results are indicated in green.

We next sought to validate our results in an independent dataset. The immunopeptidomics and proteomics datasets from the breast cancer cell line MDA-MB-231 (28) were appropriate for comparison as these are cancer cells that had also been treated with IFNγ for 48 hours (Fig. 4A). We applied the same MIP versus MDP analysis method to 9-mers. This confirmed the underrepresentation of proline at positions 4–6 and 8 of MIPs, and also showed underrepresentation in positions 2 and 3 (Fig. 4B). Plotting the proline abundance between MIPs and MDPs, and statistical analysis with the Fisher exact test, showed statistically significant differences in position s1–6 and 8–9 (Fig. 4C). We again observed a modest overrepresentation of lysine and arginine in position 1 of MIPs; however, this did not reach the Z-score threshold (Fig. 4B).

When the UEP versus IEP analysis was applied, it showed an underrepresentation of proline in positions 1–9 in IEPs (Fig. 4D), but with only position 4–6 crossing the Z-score threshold with scores of −5, −3, and −3.8 respectively. This could be due to the small sample number of 96 UEPs compared with 10,699 IEPs. Overall, the validation of an underrepresentation of proline in MIPs strongly supports that peptides harboring proline residues in positions 4, 5, and possibly also 6–8, are specifically downregulated through IFNγ.

Proline has a unique impact on peptide secondary structures; its cyclic side chain gives the amino acid conformational rigidity, inducing a “kink” of the amino acid sequence away from the proline residue which destabilizes secondary structures like alpha helices and beta-pleated sheets (48, 49). We therefore investigated whether proline substitution impacts peptide affinity to, or stability with, its associated HLA.

**FIGURE 5:**
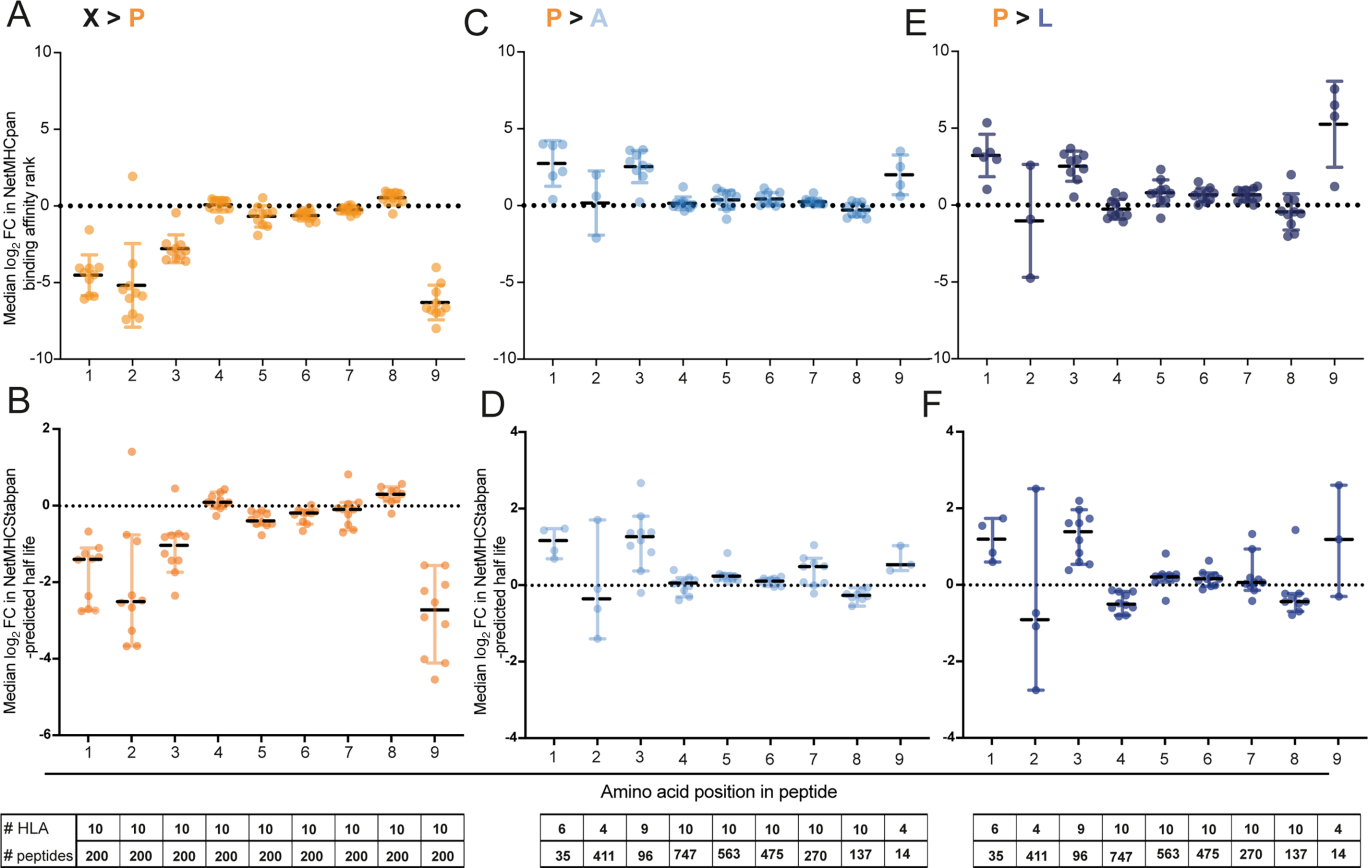
Simulating the impact of amino acid replacements on peptide affinity and binding stability to their cognate HLAs. **A,** Median log_2_ FC value from each HLA-A and -B from each PDO, demonstrating the impact of individually exchanging every single amino acid from detected peptides in positions 1–9 with proline, on NetMHCpan4.1-predicted BA rank (*N* = 200 peptides per HLA). **B,** Median log_2_ FC value from each HLA-A and -B from each PDO, demonstrating the impact of individually exchanging every single amino acid from detected peptides in positions 1–9 with proline, on NetMHCStabpan1.0-predicted (*N* = 200 peptides per HLA). **C,** Median log_2_ FC value of each HLA-A and -B from each PDO, demonstrating the impact of exchanging proline, in detected peptides with a single proline, with alanine, on NetMHCpan4.1-predicted BA rank (inidividual sample numbers annontated on the plots). **D,** Median log_2_ FC value of each HLA-A and -B from each PDO, demonstrating the impact of exchanging proline, in detected peptides with a single proline, with alanine, on on NetMHCStabpan1.0-predicted half life (inidividual sample numbers annontated on the plots). **E,** Median log_2_ FC value of each HLA-A and -B from each PDO, demonstrating the impact of exchanging proline, in detected peptides with a single proline, with leucine (inidividual sample numbers annontated on the plots), on NetMHCpan4.1-predicted BA rank. **F,** Median log_2_ FC value of each HLA-A and -B from each PDO, demonstrating the impact of exchanging proline, in detected peptides with a single proline, with leucine (inidividual sample numbers annontated on the plots), on NetMHCStabpan1.0-predicted.

We first assessed whether proline in position 4–8 influences the NetMHCpan4.1-predicted BA to HLAs or the NetMHCstabpan1.0-predicted binding stability. Although BA and stability are linked, they can differ, and stability may be more important for recognition by TCRs (50). We sequentially replaced every amino acid in turn with proline, in a sample of 200 randomly selected 9-mer MS-detected peptides from our PDOs, to investigate the impact proline inclusion has on the NetMHCpan4.1-predicted affinity and NetMHCstabpan1.0-predicted stability (Fig. 5A and B). Replacing amino acids with proline was disadvantageous for peptide-HLA BA and stability in the anchor residue positions P1–3 and P9. One exception was in the peptides which bind HLA-B35.08 in P2, where proline acts as an anchor residue, which saw increased affinity and stability. Substituting amino acids in P4–8 with proline had no strong effect on predicted BA or stability, suggesting there is low specificity in these positions for peptide-HLA binding. To scrutinize this further, we selected all peptides containing a single proline and replaced it with either alanine or leucine (Fig. 5C–F). Alanine was selected as it eliminates side chain interactions and does not distort the confirmation of the main chain like proline. Leucine has similar properties to alanine, but it is a larger amino acid, so it is used where maintaining amino acid size may be important. Proline was infrequently detected in P1, 3, and 9 as it is less well tolerated in anchor positions for most HLA-I. The exception was again HLA-B35.08, which provides 98% of the detected peptides with proline in P2, due to its preference for proline as the anchor residue, for which replacement of proline in P2 led to a large decrease in BA and stability. Only small changes in peptide-HLA BA and stability are seen in when replacing proline with alanine or leucine in P4–8; suggesting that proline in these positions does not cause any notable structural changes, and does not influence the affinity or stability of the peptides for their predicted HLA.

## Discussion

This study shows that peptide remodeling through IFNγ is complex and influenced by multiple distinct mechanisms. Upregulation or downregulation of proteins by IFNγ largely showed concordant abundance changes of corresponding peptides. However, an even larger fraction of peptides changed in cell surface abundance despite rather stable protein abundance. We and others have previously shown that this can be attributed, in part, to the increased chymotryptic activity of the immunoproteasome (27, 30). Furthermore, IFNγ signaling disproportionally increased HLA-B compared with HLA-A surface expression which led to an increase in peptides presented on HLA-B. Moreover, demonstrating that even peptides originating from the same protein can show a mix of upregulation and downregulation, and that this is neither dependent on their HLA binding affinities, nor their location within the source protein, allowed us to isolate peptide-specific characteristics that affect their abundance in IFNγ conditions.

The most notable novel finding was the underrepresentation of proline in the core of MIPs and IEPs. It has previously been shown that proline in the core of the peptide sequence protects peptides from internal cleavage by endopeptidases and the proteasome (46, 51). Known proline endopeptidases are DPP9, PREP, DPP8, DPP3, but these were not upregulated by IFNγ in our data. However, it is possible that the activity of such peptidases increases. An alternative theory is that the protective effect of proline is more relevant in the absence of IFNγ. Studies have shown that up to 99% of all proteasome-generated peptides are degraded by peptidases (52). In the untreated condition, abundance and activity of the antigen processing and presentation machinery, alongside expression and supply of peptide-receptive HLA are the limiting factors in surface HLA presentation (41). The result is that peptides may spend more time in the cytoplasm and endosplasmic reticulum before being shuttled to the cell surface, giving any single peptide more exposure to peptidases and hence, a higher probability of internal cleavage. Acceleration of peptide generation, processing, and rapid loading onto HLA-I through IFNγ may allow peptides without proline to escape degradation. This would then dilute proline-containing peptides within the peptide pool and explain their relative decrease or drop out.

Our insights into determinants of peptide abundance changes with IFNγ exposure could be useful to improve the design of cancer vaccines or TCR engineered therapies as it could enable the more accurate selection of peptides likely to be presented on patient tumors. For example, peptides presented on HLA-B, which are likely to increase in intensity when T cells release IFNγ, may be preferable targets over those presented by HLA-A. Peptides with proline in positions 4 and 5, which favors peptide downregulation or even makes them undetectable in cancer cells exposed to IFNγ, can be avoided. Whether the overall increase in HLA surface expression, or the remodeling of the immunopeptidome is more relevant for the critical role of IFNγ signaling for immunotherapy responses needs to be further investigated.

## Supplementary Material

Data CRC01 RNA, protein and peptide data for CRC01

Data CRC04 RNA, protein and peptide data for CRC04

Data CRC05 RNA, protein and peptide data for CRC05

Supplementary Figure and Table Legends Supplementary Figure and Table Legends 1-2

Supplementary Table 1 Supplementary Table 1. Statistics summary from our peptides which most increase (MIPs) and decrease in intensity (MDPs), derived from proteins with a -1 to +1 log2 fold change, separated by NetMHCpan4.1b-attributed HLA: unique peptide count, distribution and mean and median of allotype-normalized immunopeptidomics FC.

Supplementary Table 2 Supplementary Table 2. Unique peptide count for untreated-exclusive peptides (UEPs) and IFNg-exclusive peptides(IEPs) from our 3 CRC PDOs, separated by NetMHCpan4.1b-attributed HLA.

Supplementary Figure 1 Supplementary Figure 1. Effect of relative peptide position within protein on peptide abundance changes under IFNγ treatment.

Supplementary Figure 2 Supplemental Figure 2. Additional internal validation of difference in amino acid features of peptides between untreated and IFNγ-treated conditions.
