# Supplementary material for: Multifactorial Remodeling of the Cancer Immunopeptidome by IFNγ
Source: Cancer Res Commun. 2023 Nov 17;3(11):2345–57. doi: 10.1158/2767-9764.CRC-23-0121 (PMC10655636; doi:10.1158/2767-9764.CRC-23-0121)
Supplement: Supplementary Figure and Table Legends — 1-2 [file crc-23-0121-s04.pdf]

Supplementary material for Newey et al.

Table of contents:

Supplementary Table 1: *Summary statistics of most increasing and most decreasing peptides.* ***Page 2***

Supplementary Table 2: *Unique peptide count for untreated-exclusive peptides and IFNγ-exclusive peptides.* ***Page 3***

Supplementary Figure 1: *Effect of relative peptide position within protein on peptide abundance changes under IFNγ treatment.* ***Page 4-6***

Supplementary Figure 2: *Additional internal validation of difference in amino acid features of peptides between untreated and IFNγ-treated conditions* ***Page 7-8***
