# Supplementary material for: Multifactorial Remodeling of the Cancer Immunopeptidome by IFNγ
Source: Cancer Res Commun. 2023 Nov 17;3(11):2345–57. doi: 10.1158/2767-9764.CRC-23-0121 (PMC10655636; doi:10.1158/2767-9764.CRC-23-0121)
Supplement: Supplementary Table 1 — Statistics summary from our peptides which most increase (MIPs) and decrease in intensity (MDPs), derived from proteins with a -1 to +1 log2 fold change, separated by NetMHCpan4.1b-attributed HLA: unique peptide count, distribution and mean and median of allotype-normalized immunopeptidomics FC. [file crc-23-0121-s05.pdf]

Supplemental tables

Supplementary table 1. Statistics summary from our peptides which most increase (MIPs) and decrease in intensity (MDPs), derived from proteins with a -1 to +1 log2 fold change, separated by NetMHCpan4.1b-attributed HLA: unique peptide count, distribution and mean and median of allotype-normalized immunopeptidomics FC.

Category	PDO line	Source HLA	Peptide count	1st quartile	Median	Mean	3rd quartile
MIP	CRC-01	HLA.A31.01	199	8.22	11.35	41.92	18.89
MIP	CRC-01	HLA.A32.01	125	10.08	15.28	34.32	24.75
MIP	CRC-01	HLA.B14.01	116	14.56	18.74	30.45	33.20
MIP	CRC-01	HLA.B27.05	98	12.82	19.23	33.88	33.39
MIP	CRC-01	HLA.C02.02	25	8.99	13.31	27.31	16.97
MIP	CRC-01	HLA.C08.02	57	6.81	9.58	12.84	13.63
MIP	CRC-04	HLA.A03.01	184	2.32	3.09	11.54	4.96
MIP	CRC-04	HLA.A24.02	79	2.34	3.88	13.93	10.65
MIP	CRC-04	HLA.B18.01	31	2.78	3.61	13.01	8.19
MIP	CRC-04	HLA.B35.08	56	3.26	5.03	40.67	10.33
MIP	CRC-04	HLA.C04.01	11	12.16	14.61	23.21	27.71
MIP	CRC-04	HLA.C05.01	10	3.61	4.58	5.21	6.38
MIP	CRC-05	HLA.A32.01	37	16.71	27.71	35.17	33.84
MIP	CRC-05	HLA.B40.01	90	12.86	17.62	36.11	24.89
MIP	CRC-05	HLA.C03.04	37	15.70	19.13	28.91	28.35
MDP	CRC-01	HLA.A31.01	197	0.21	0.29	0.28	0.37
MDP	CRC-01	HLA.A32.01	125	0.27	0.34	0.33	0.42
MDP	CRC-01	HLA.B14.01	115	0.30	0.38	0.36	0.46
MDP	CRC-01	HLA.B27.05	98	0.29	0.41	0.40	0.53
MDP	CRC-01	HLA.C02.02	24	0.19	0.22	0.24	0.32
MDP	CRC-01	HLA.C08.02	57	0.17	0.28	0.25	0.34
MDP	CRC-04	HLA.A03.01	182	0.09	0.14	0.13	0.18
MDP	CRC-04	HLA.A24.02	78	0.07	0.13	0.12	0.16
MDP	CRC-04	HLA.B18.01	31	0.13	0.19	0.17	0.21
MDP	CRC-04	HLA.B35.08	56	0.11	0.19	0.16	0.21
MDP	CRC-04	HLA.C04.01	12	0.12	0.14	0.14	0.16
MDP	CRC-04	HLA.C05.01	10	0.24	0.27	0.27	0.30
MDP	CRC-05	HLA.A32.01	37	0.41	0.63	0.60	0.77
MDP	CRC-05	HLA.B40.01	90	0.34	0.66	0.61	0.90
MDP	CRC-05	HLA.C03.04	37	0.31	0.52	0.49	0.70
