# Supplementary material for: Multifactorial Remodeling of the Cancer Immunopeptidome by IFNγ
Source: Cancer Res Commun. 2023 Nov 17;3(11):2345–57. doi: 10.1158/2767-9764.CRC-23-0121 (PMC10655636; doi:10.1158/2767-9764.CRC-23-0121)
Supplement: Supplementary Table 2 — Unique peptide count for untreated-exclusive peptides (UEPs) and IFNg-exclusive peptides(IEPs) from our 3 CRC PDOs, separated by NetMHCpan4.1b-attributed HLA. [file crc-23-0121-s06.pdf]

Supplementary table 2. Unique peptide count for untreated-exclusive peptides (UEPs) and IFNγ-exclusive peptides (IEPs) from our 3 CRC PDOs, separated by NetMHCpan4.1b-attributed HLA.

	**UEPs**	**IEPs**
CRC-01 HLA.A31.01	166	186
CRC-01 HLA.A32.01	97	136
CRC-01 HLA.B14.01	42	212
CRC-01 HLA.B27.05	25	234
CRC-01 HLA.C02.02	18	33
CRC-01 HLA.C08.02	95	113
CRC-04 HLA.A03.01	231	305
CRC-04 HLA.A24.02	155	119
CRC-04 HLA.B18.01	73	91
CRC-04 HLA.B35.08	141	126
CRC-04 HLA.C04.01	43	60
CRC-04 HLA.C05.01	25	63
CRC-05 HLA.A32.01	38	60
CRC-05 HLA.B40.01	52	175
CRC-05 HLA.C03.04	28	51
