# Supplementary material for: Multifactorial Remodeling of the Cancer Immunopeptidome by IFNγ
Source: Cancer Res Commun. 2023 Nov 17;3(11):2345–57. doi: 10.1158/2767-9764.CRC-23-0121 (PMC10655636; doi:10.1158/2767-9764.CRC-23-0121)
Supplement: Supplementary Figure 1 — Effect of relative peptide position within protein on peptide abundance changes under IFNγ treatment. [file crc-23-0121-s07.pdf]

Supplementary Figure 1

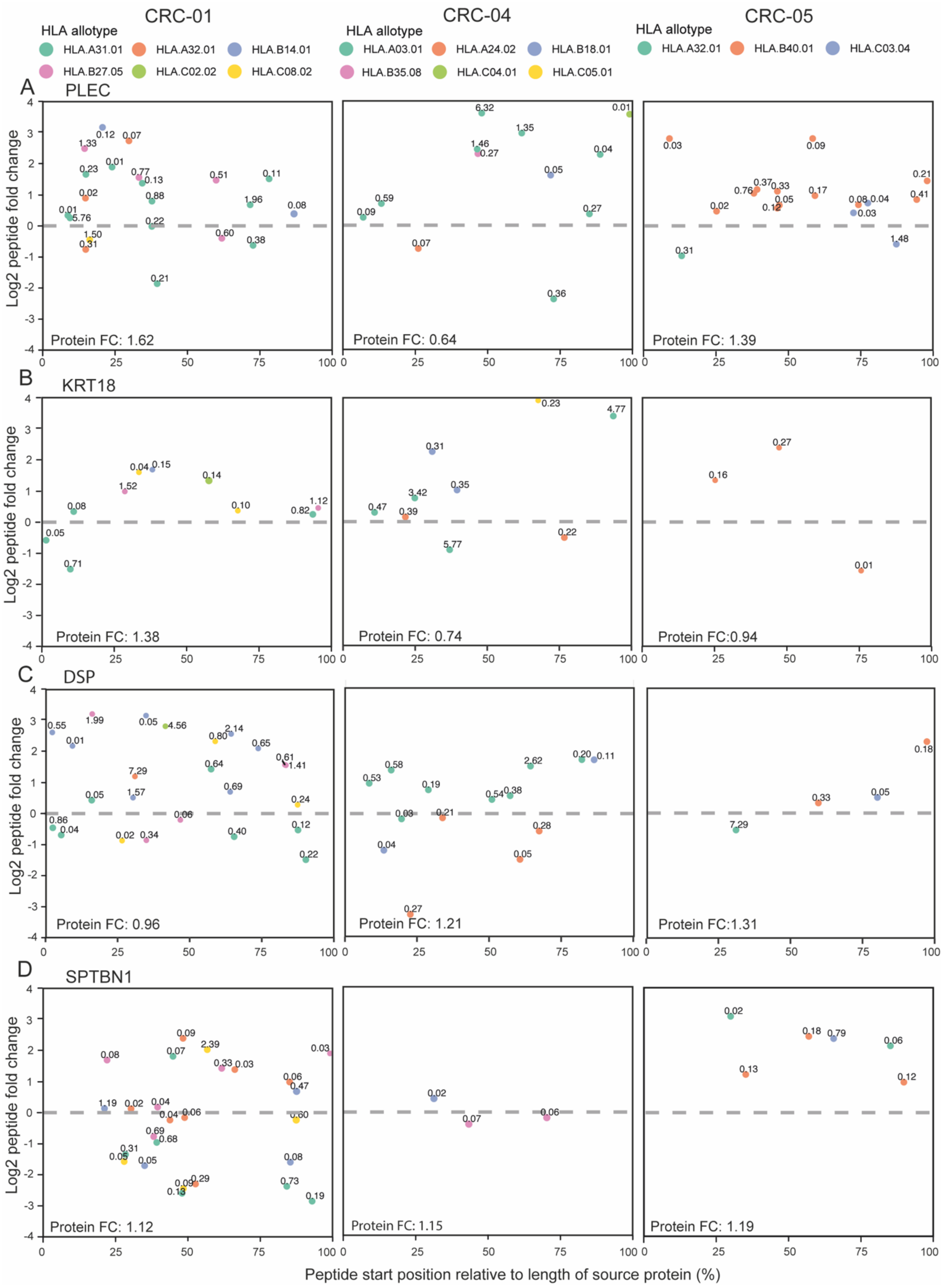

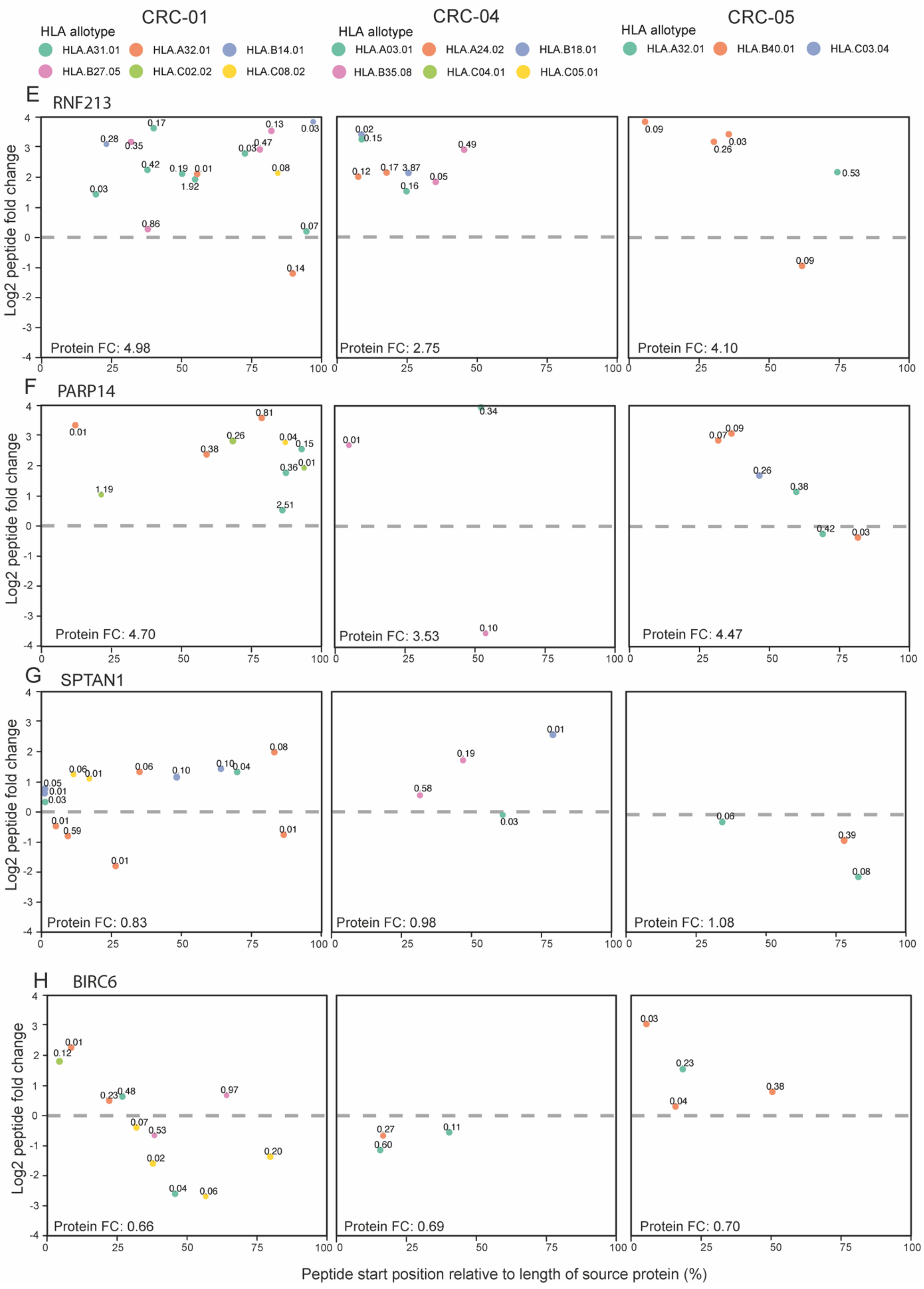
CRC-01
HLA allotype
HLA.A31.01
HLA.A32.01
HLA.B14.01
HLA.B27.05
HLA.C02.02
HLA.C08.02
CRC-04
HLA allotype
HLA.A03.01
HLA.A24.02
HLA.B18.01
HLA.B35.08
HLA.C04.01
HLA.C05.01
CRC-05
HLA allotype
HLA.A32.01
HLA.B40.01
HLA.C03.04
E
RNF213
Protein FC: 4.98
Protein FC: 2.75
Protein FC: 4.10
F
PARP14
Protein FC: 4.70
Protein FC: 3.53
Protein FC: 4.47
G
SPTAN1
Protein FC: 0.83
Protein FC: 0.98
Protein FC: 1.08
H
BIRC6
Protein FC: 0.66
Protein FC: 0.69
Protein FC: 0.70
Log2 peptide fold change
Peptide start position relative to length of source protein (%)

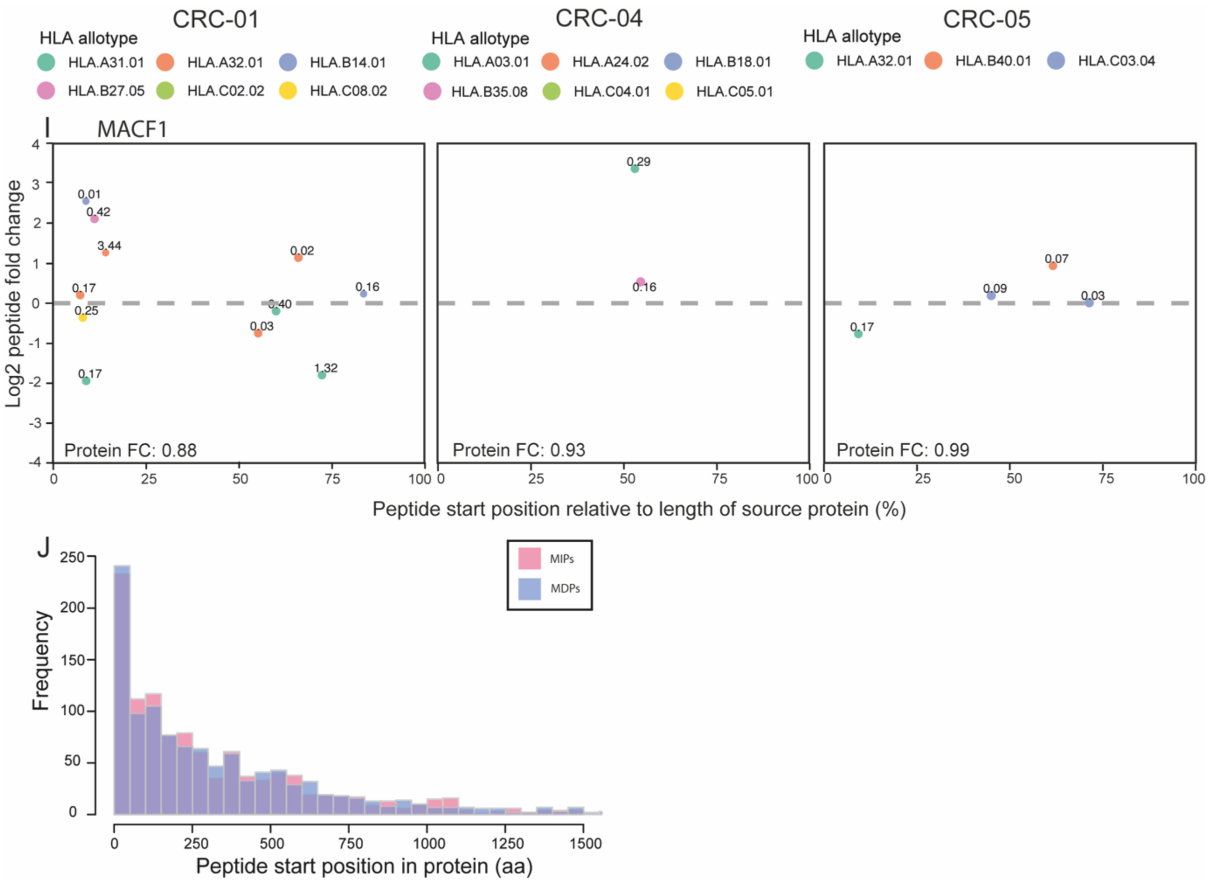

Supplemental figure 1*. Effect of relative peptide position within protein on peptide abundance changes under IFNγ treatment.* **A-I:** Log2 change in peptide intensity between untreated and IFNγ conditions for peptides derived from the single source protein, plotted against the relative position of the peptide in protein. Long proteins with the most peptides across the 3 PDOs selected. Points color-coded by their NetMHCpan4.1-predicted source HLA, with the NetMHCpan4.1 predicted binding affinity rank annotated above. Protein fold change for the source protein in each organoid noted at the bottom of each plot. PLEC, KRT18, DSP, SPTBN1, RNF213, PARP14, SPTAN1, BIRC6, MACF1. **J:** Frequency distribution of peptide absolute start positions for all MIPs against all MDPs.
