# Supplementary material for: Multifactorial Remodeling of the Cancer Immunopeptidome by IFNγ
Source: Cancer Res Commun. 2023 Nov 17;3(11):2345–57. doi: 10.1158/2767-9764.CRC-23-0121 (PMC10655636; doi:10.1158/2767-9764.CRC-23-0121)
Supplement: Supplementary Figure 2 — Supplemental Figure 2. Additional internal validation of difference in amino acid features of peptides between untreated and IFNγ-treated conditions. [file crc-23-0121-s08.pdf]

Supplementary Figure 2

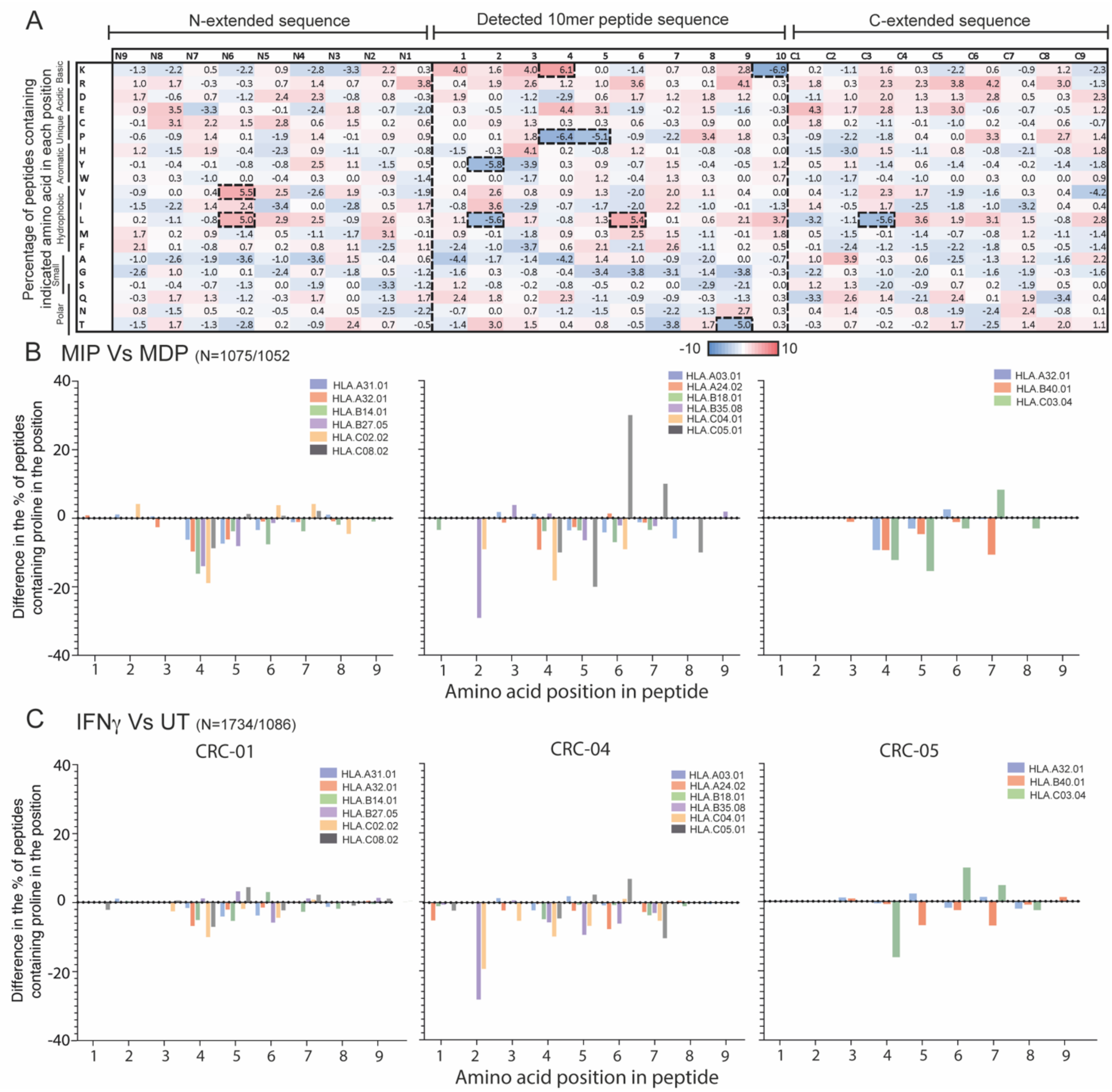

Supplemental figure 2. ***Additional internal validation of difference in amino acid features of peptides between untreated and IFNγ-treated conditions.*** **A:** Heatmap of the amino acid composition changes between 10-mer most increasing peptides (MIPs) and most decreasing peptides (MDPs), alongside N- and C extensions. Peptides were selected from ‘low fold-change’ source proteins (0.5-2X fold-change) then grouped by their NetMHCpan4.1-predicted HLA-I allotype, then from each group the top 10th percentile peptide FC (MIPs) and bottom 10th percentile peptide FC (MDPs) were selected. Percentage of peptides with highlighted amino acid in each position were calculated for each group, then the percentage values for the MDPs (N=333) were subtracted from the MIPs (N=330). **B:** A graph depicting the change in the percentage of

peptides with proline in each position between MIPs and MDPs, split by PDO and by NetMHCpan4.1-predicted source HLA (peptide numbers detailed in Supplementary Table 1 and 2). **C:** A graph depicting the change in the percentage of peptides with proline in each position between IFNγ-exclusive peptides (IEPs) and untreated-exclusive peptides (UEPs), split by PDO and by NetMHCpan4.1-predicted source HLA (peptide numbers detailed in Supplementary Table 3).
